# Physical Disease: A Crucial Research Area in the Context of Quality of Life and Mental Health

**DOI:** 10.3390/children10061062

**Published:** 2023-06-15

**Authors:** Paraskevi Theofilou

**Affiliations:** 1 General Hospital of Thoracic Diseases Sotiria, 115 27 Athens, Greece; pardrothe@gmail.com; 2 School of Social Sciences, Hellenic Open University, 263 35 Patra, Greece; 3 Lab of Experimental and Applied Psychology, SCG-Scientific College of Greece, 106 73 Athens, Greece

The medical literature defines a chronic physical disease as any organic disorder that lasts more than three months or that entails a period of hospitalization of more than of a month, which restricts an individual’s daily abilities or behaviors and causes changes to their social functions [1]. Therefore, the criteria by which a disease is defined as chronic is the temporal duration of the disease, the degree of its severity, the extent to which it affects the functionality of the individual and the need it creates for lasting care from health services.

Specifically, it seems that chronic physical diseases incidence rates are notably increased among children and adolescents. Epidemiological studies, conducted in the USA and in England, have estimated that about 10–15% of all children will present a chronic disease [2]. Chronic physical illness takes a toll on childhood, with organic and functional problems, repeated medical visits, complex examinations, frequent hospitalizations, uncertainty about the future and complex secondary psychological, social and educational problems. Because of the multiple issues it creates, chronic disease is considered a source of long-term stress for the child and their family. The responsibility of treating the disease is shared between the doctor, the child and the family.

Life after the diagnosis of a chronic physical disease is very stressful and is only the beginning of the adaptation process. Tomorrow looks doubtful and any plan for the future seems meaningless. The child and their family do not have to cope alone with the changes arising from the course of the disease and its symptoms, as well as the psychological and social changes which follow [3].

Undoubtedly, chronic diseases hugely affect the everyday functionality and mental well-being of a child in all aspects of their life. For this reason, the comprehensive treatment of the effects of chronic physical illness, both medically and on a psychosocioeconomic level, requires the provision of comprehensive care through coordinated medical, psychological, educational and social services [3]. The state must stand by its citizens and promote the social awareness.

The care provided to the child needs to be family-oriented because the effects of the disease are shared by the family [4]. Additionally, it is useful to rely on community-based services to avoid the psychological effects of powerlessness and the inadequacy they bring to children upon frequent visits to hospitals. It is therefore understandable that the close cooperation of the health services and the contribution of the agencies active in the fight against risk factors to chronic disease is necessary for the successful implementation of specific effective actions. At the same time, it is advisable that combine their work with other methods, such as communication, education, legislation, financial measures, organizational changes and community socio-economic development.
